# Raman Spectra and Bulk Modulus of Nanodiamond in a Size Interval of 2–5 nm

**DOI:** 10.1186/s11671-017-2333-0

**Published:** 2017-10-10

**Authors:** Mikhail Popov, Valentin Churkin, Alexey Kirichenko, Viktor Denisov, Danila Ovsyannikov, Boris Kulnitskiy, Igor Perezhogin, Viktor Aksenenkov, Vladimir Blank

**Affiliations:** 1 Technological Institute for Superhard and Novel Carbon Materials, Centralnaya str. 7a, Troitsk, Moscow, Russian Federation 142190; 2 National University of Science and Technology MISiS, Leninskiy prospekt 4, Moscow, Russian Federation 119049; 3 Moscow Institute of Physics and Technology State University, Institutskiy per. 9, Dolgoprudny, Moscow Region, Russian Federation 141700; 4 M.V.Lomonosov Moscow State University, Leninskie Gory 1, Moscow, Russian Federation 119991; 5 Institute of spectroscopy RAS, Fizicheskaya Str. 5, Troitsk, Moscow, Russian Federation 108840

**Keywords:** Nanodiamond, Raman Spectroscopy, High Pressure

## Abstract

Nanodiamond in a 2–5-nm size interval (which is typical for an appearance of quantum confinement effect) show Raman spectra composed of 3 bands at 1325, 1600, and 1500 cm^−1^ (at the 458-nm laser excitation) which shifts to 1630 cm^−1^ at the 257-nm laser excitation. Contrary to sp^2^-bonded carbon, relative intensities of the bands do not depend on the 458- and 257-nm excitation wavelengths, and a halfwidth and the intensity of the 1600 cm^−1^ band does not change visibly under pressure at least up to 50 GPa. Bulk modulus of the 2–5-nm nanodiamond determined from the high-pressure study is around 560 GPa. Studied 2–5-nm nanodiamond was purified from contamination layers and dispersed in Si or NaCl.

## Background

Nanodiamond properties’ studies have attracted high interest of researchers during the past 30 years [1]. Meanwhile, an important aspect of a quantum confinement effect influence on the mechanical properties and Raman spectra of diamond nanocrystals is practically omitted. A typical length scale for an appearance of quantum confinement effect is the Bohr radius of excitons [2]; the Bohr radius of exciton for diamond is 1.57 nm which is appropriate for the nanocrystal size of around 3 nm. Data of a parallel electron energy loss spectroscopy (PEELS) [3] provide more distinctive range for the size below 5 nm where new properties related to a modification of bonds in the nanodiamond appear. Nanodiamonds with non-modified surfaces of a size below 2 nm are not stable [1, 3] which restricts the size interval of nanodiamonds studied here by a 2–5-nm range.

According to both PEELS and nuclear magnetic resonance (NMR) spectroscopy data [3, 4], there is no sp^2^-bonded carbon in nanodiamond. As a result of quantum confinement effect, a bandgap of nanodiamond increases in the size interval of 2–5 nm, along with discrete energy levels arising at the band edges [1, 5]. In a case of covalently bonded solids, the bandgap growth means an increase in the chemical bond energy which means an increase in elastic moduli [6]. Indeed, the increase of bulk modulus to 500 GPa was derived from a pressure-volume relationship of nanodiamond [7]. Meanwhile, lattice parameters of nanodiamond including the 2–5-nm size interval correspond to those of natural diamond [8].

Raman spectra of nanodiamond are summarized in a review by Mochalin et al. [1]. Due to phonon confinement effect, a triple-degenerated Raman band at 1333 cm^−1^ of bulk diamond crystal shifts to 1325 cm^−1^ in the 2–5-nm nanodiamond. In addition, a shoulder around 1250 cm^−1^ and bands at 1590, 1640, and 1740 cm^−1^ appear in Raman spectra of nanodiamond. A set of the 1590–1740 cm^−1^ bands is attributed to sp^2^-carbon (which, as mentioned above, is absent in nanodiamond), O-H and C=O groups [1]. A relative intensity of the bands at 1325 cm^−1^ and around 1600 cm^−1^ depends on purification of nanodiamond. To avoid luminescence, spectra were usually recorded using the 325-nm laser excitation.

In the above assignment of Raman bands 1325 cm^−1^ to sp^3^- and 1600 cm^−1^ to sp^2^-bonded carbon, there is a self-contradiction related to a resonant Raman scattering effect. A scattering cross-section of sp^2^-bonded carbon exceeds the one of sp^3^-bonded carbon by a factor of 50–200 at the laser excitation in the visible range, and the cross-sections are mutually equal at the 257-nm laser excitation [9]. We have revealed in our study that relative intensities of Raman bands at 1325 and 1600 cm^−1^ of the 2–5-nm nanodiamond purified from contamination layers do not depend on the excitation wavelength in the 257–532-nm range. We observed an addition Raman band which shifts from 1500 cm^−1^ at the 458-nm laser excitation to 1630 cm^−1^ at the 257-nm laser excitation. Bulk modulus of the 2–5-nm nanodiamond estimated in our study is around 560 GPa.

## Methods

We used a detonation 2–5-nm diamond produced by the SINTA company (Republic of Belarus). For removing the rest of the contamination layers, the 2–5-nm nanodiamond was treated in a planetary mill with a mixture of 25 wt% of Si or NaCl. A Fritsch planetary mill with ceramic silicon nitride (Si_3_N_4_) bowls and balls of 10 mm in diameter was used. The treatment in the planetary mill provides preparation of homogeneous nanocomposites without contamination by material of the balls [10–12].

We also used a nanodiamond water suspension with a mean crystal size of 25 nm produced at the Microdiamant AG (MSY Liquid Diamond product; MSY diamond is a monocrystalline diamond powder produced by HPHT (high-pressure, high-temperature) synthesis) for high-pressure study. All structure studies of the 25-nm nanodiamond were done after drying the suspension.

The Raman spectra were recorded with a TRIAX 552 (Jobin Yvon Inc., Edison, NJ) spectrometer, equipped with a CCD Spec-10, 2KBUV Princeton Instruments 2048 × 512 detector and razor edge filters. Transmission electron microscope (TEM) and X-ray studies were done by a JEM 2010 high-resolution microscope (JEOL Ltd., Tokyo, Japan) and Empyrean (PANalytical) X-ray diffractometer. We used a diamond anvil cell (DAC) for a high-pressure study. The pressure was measured from the stress-induced shifts of the Raman spectra from the diamond anvil [13].

**Fig. 1:**
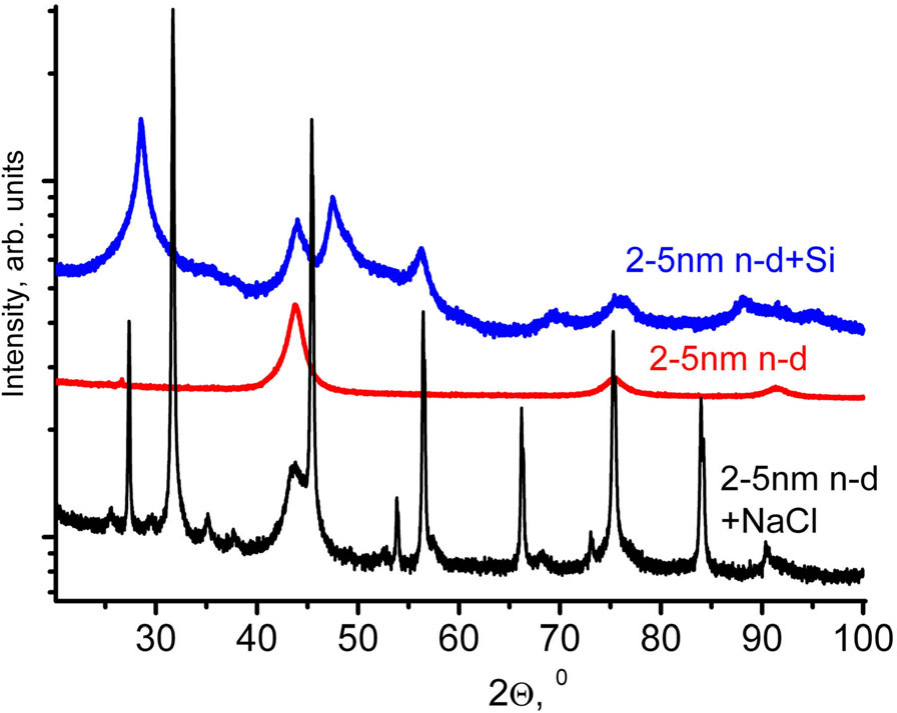
The X-ray powder diffraction (XRD) spectra of initial 2–5-nm nanodiamond (2–5 n-d) and 2–5-nm nanodiamond treated in a planetary mill with a mixture of 25 wt% of Si (2–5 n-d + Si) and NaCl (2–5 n-d + NaCl)

The X-ray powder diffraction (XRD) (Fig. 1) spectra were treated using the MAUD program and the Rietveld refinement method. The calculated mean crystal size is around 5 nm. A diffraction band (400) (2*θ* around 120°) appropriated to an interplanar distance *d*_400_ = 0.892 Å was used for the lattice parameter calculation which equals to 3.567 ± 0.002 Å. Thus, the lattice parameter of the 2–5-nm nanodiamond used in our study corresponds to that of natural diamond.

**Fig. 2:**
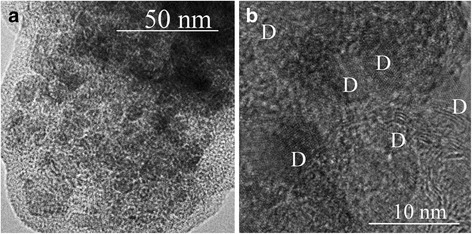
TEM images of nanodiamond mixed with Si after the planetary mill treatment. The nanodiamond grains are separated by disordered Si. The grain size lies in the range of 2–5 nm. **a** General view. (**b**) High-resolution image. Nanodiamond grains are market by D in **b**

TEM images of nanodiamond mixed with Si after the planetary mill treatment are shown in Fig. 2. The nanodiamond grains are separated by disordered Si. The grain size lies in the range of 2–5 nm.

## Results and Discussion

**Fig. 3:**
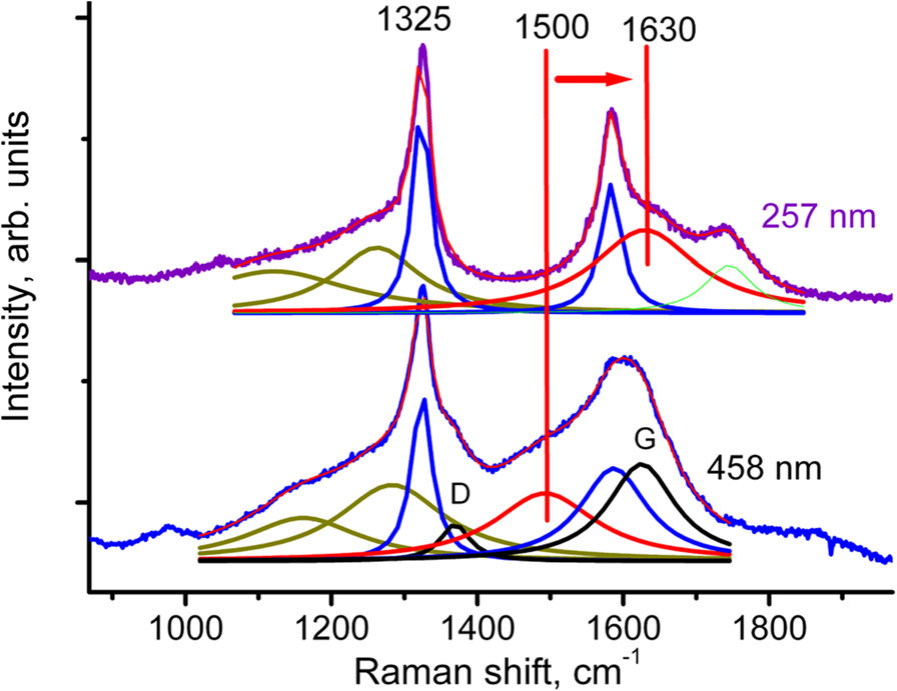
Raman spectra of the 2–5-nm nanodiamond at the 257 and 458-nm excitation wavelengths. Raman spectra compose of peaks at 1325 cm^−1^ (with the shoulder around 1250 cm^−1^), 1600 cm^−1^ and the 1500 cm^−1^ band observed at 458 nm which shifts to 1630 cm^−1^ at 257 nm. In addition, sp^2^-bonded pollution at 1360 and 1620 cm^−1^ (D and G bands) are present in the spectra. Lorentz multi-peaks fits are plotted

Raman spectra of the 2–5-nm nanodiamond are plotted in Fig. 3. There is no dependence of Raman spectra upon the preparation of the 2–5-nm nanodiamond samples (powder or mixture with NaCl or Si). The intensity of a laser beam was minimized down to a level (a typical laser beam power was 0.7 mW focused in a 2-μm spot) when a possible heating of the samples did not lead to any visible downshifts of the Raman bands. In the case of the mixture with Si, increasing of the laser power (to 7 mW focused in a 2-μm spot) have led to an appearance of SiC bands in Raman spectra along with disappearance of the diamond band. The SiC creation means the absence of contaminations in the boundaries between nanodiamond and Si and indicates that the treatment in the planetary mill removes the groups composed of different combinations of C, O, N, H from the nanodiamond surfaces [1], but the contaminations stay in a stuff (Si or NaCl). Thus, the band at 1740 cm^−1^ (one is visible more distinctly at 257-nm excitation) of the contaminations groups is present in the Raman spectra (Fig. 3). The 1740 cm^−1^ band is assigned to C=O band of functional groups (possibly from carboxylic groups (− COOH)) [14].

The resonance behavior of the band at 1600 cm^−1^ under the 458 nm and 257 nm laser excitations was not observed: the intensity of the band is the same at both excitations. Raman spectra at the 458-nm excitation include the peaks at 1325 cm^−1^ (with the shoulder around 1250 cm^−1^), 1500 cm^−1^, and 1600 cm^−1^. In addition, sp^2^-bonded pollution at 1360 and 1620 cm^−1^ (D and G bands) are present in the spectra.

Lorentz multi-peaks fits are plotted in Fig. 3. Raman spectra at the 257-nm excitation consist of the same peaks 1325 cm^−1^ (with shoulder around 1250 cm^−1^) and 1600 cm^−1^. The D and G bands of the pollution disappeared from the spectra, because the Raman scattering cross-section of sp^2^-bonded carbon decreases by the factor of 50–200 upon changing the excitation wavelength from 458 to 257 nm as mentioned above. The band around 1500 cm^−1^ shifts to 1630 cm^−1^. The observed resonance shift (dispersion) of the band around 1500 to 1630 cm^−1^ is typical for different carbon clusters with conjugated bonds where carbon atoms have 3 and 4 neighbors (for example, 3D C_60_, ultrahard fullerite or diamond-like carbon) [15–17]. In Ref. [18], the resonant Raman spectra of tetrahedral amorphous carbon were calculated and the dispersion of the band around 1500 cm^−1^ was attributed to a presence of sp^2^ chains. Nevertheless, no chains are expected in nanodiamond; there are no place for sp^2^ chains in a structure of 3D C_60_, and no chains were observed in ultrahard fullerite. Thus, the reason of the dispersion in the last group of carbon clusters is not clear.

**Fig. 4:**
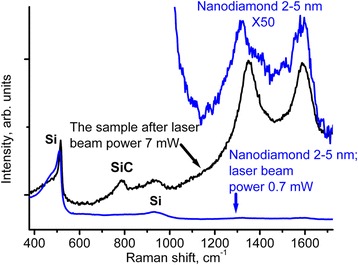
Raman spectra of the 2–5-nm nanodiamond mixed with Si (the bottom spectrum) and created after high-power irradiation sp^2^ clusters (the middle spectrum). The upper spectrum appropriates to the bottom spectrum with the intensity multiplied by the factor of 50. Bands related to Si (the first and the second orders) and SiC (around 790 cm^−1^) are marked. The spectra were acquired at the same 0.7 mW laser beam power. Excitation wavelength was 532 nm

The increase of the laser beam power from 0.7 to 7 mW led to the mentioned above transformation of nanodiamond 2–5 nm mixed with Si to SiC and sp^2^ carbon clusters (Fig. 4). The Raman cross-section of the created sp^2^-clusters exceeds the one of the 2–5-nm nanodiamond by a factor ~ 50 (including the 1600 cm^−1^ band). In Fig. 4, the bands related to Si (the first and the second orders) and SiC (around 790 cm^−1^) are marked. The spectra of the 2–5 nm nanodiamond (the bottom spectrum) and the created after high-power irradiation sp^2^ clusters (the middle spectrum) were acquired at the same laser beam power of 0.7 mW. The upper spectrum appropriates to the bottom spectrum with the intensity multiplied by the factor 50.

The absence of the resonance effect for the 1600 cm^−1^ band indicates an attribution of the band to phonon features of the 2–5-nm nanodiamond instead of an sp^2^-bonded fraction. Consequently, force constants appropriate to Raman bands 1333 cm^−1^ (this one is shifted to 1325 cm^−1^ because of a phonon confinement effect [1]), 1500–1630 cm^−1^, and 1600 cm^−1^ determine the elastic module of the 2–5-nm nanodiamond according to the dynamical theory of crystal lattices [19]. Typically, the Raman frequency *ω* scales upon the force constant *k* as *ω*~(*k*/*m*)^2^ where *m* is an atom mass, and the presence of the additional higher frequency bands in the Raman spectra means an increasing elastic module.

The dependence of the 2–5-nm nanodiamond Raman spectra on pressure provides information on bulk modulus. Indeed, taking into account the known relation [20]

1 $$ {\gamma}_i=-\frac{\partial \ln {\omega}_i}{\partial \ln V}=\frac{B_0}{\omega_0}\frac{\partial {\omega}_i}{\partial P} $$

where *γ*_i_ is the Gruneisen parameter for a quasiharmonic mode of frequency *ω*_i_ (*ω*_0_ marks the one at zero pressure, *B*_0_ is bulk modulus); we obtain the bulk modulus from the dependence *ω*(*P*). In general, *γ* ≈ 1 for covalently bonding group IV semiconductors [20], *γ* = 0.96 for diamond [21], and *γ* ≈ 1.1 for graphene plane [22]. For our estimations below, we use *γ* ≈ 1.

The mixture of 2–5-nm nanodiamond and NaCl (as mentioned in the “Methods” section, the 2–5-nm nanodiamond was treated in a planetary mill with a mixture of 25 wt% of NaCl) was loaded in a DAC. NaCl acts as a pressure-transmitting medium: under pressure below 50 GPa, a yield strength of NaCl varies from 0.08 to 0.65 GPa depending on pressure [23] (the strength increases upon pressure growth to 28 GPa and decreases about 50% at higher pressures). Consequently, a value of non-hydrostaticity [13] (*σ*_1_ − *σ*_2_)/*σ*_1_ (*σ*_1_ and *σ*_2_ are major stresses in the sample) is below 5%.

**Fig. 5:**
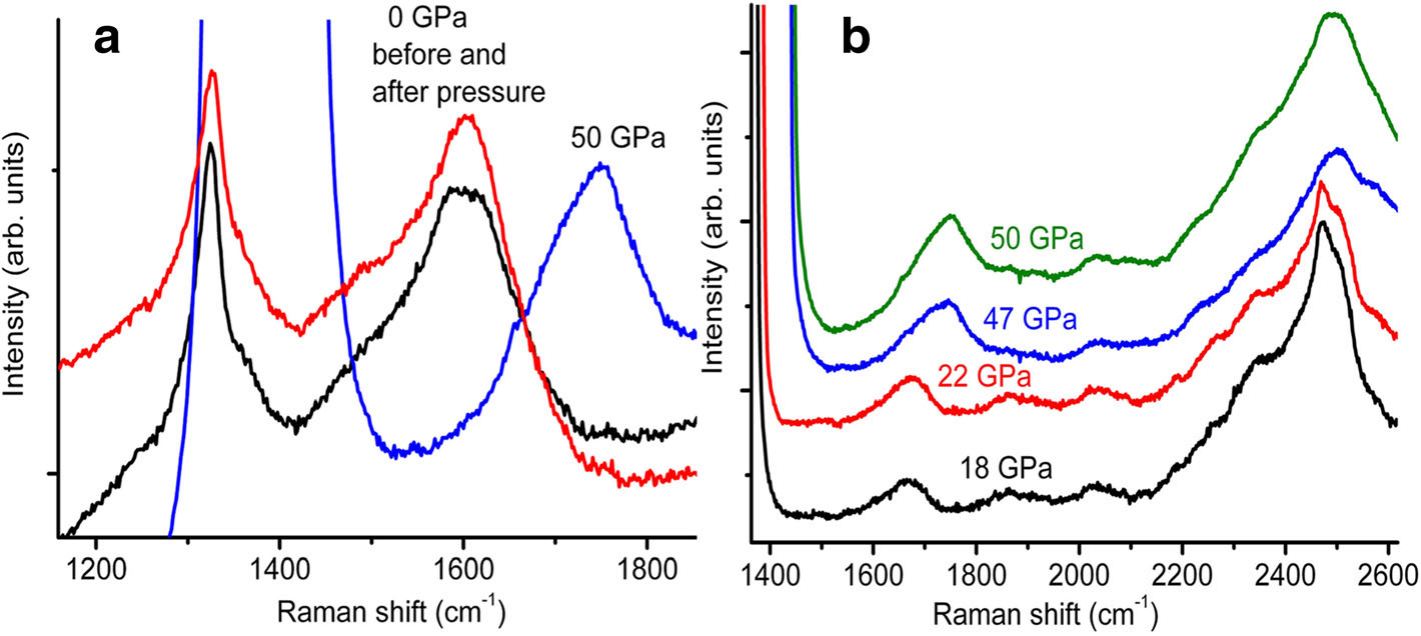
**a** Raman spectra of the 2–5-nm nanodiamond-NaCl nanocomposite before and after pressure treatment and at a 50 GPa pressure. Excitation wavelength is 458 nm. The absence of the band with *ω*_0_ = 1325 cm^−1^ under pressure of 50 GPa is possible only for a case when the bulk modulus of the 2–5-nm nanodiamond exceeds 524 GPa. (**b**) Pressure induced shift of the 1600 cm^−1^ Raman band; one halfwidth and intensity do not change under pressure

Raman spectra of the nanocomposite before and after the pressure treatment and at a 50 GPa pressure are illustrated in Fig. 5a. We did not observe any changes in Raman spectra after the pressure treatment. A halfwidth and an intensity of the 1600 cm^−1^ band did not change under pressure (Fig. 5b). This behavior of the 1600 cm^−1^ band of 2–5-nm nanodiamond distinguishes essentially from a pressure-induced transformations of G band of graphite, diamond-like carbon and glassy carbon where the halfwidth of G band increases drastically (by a factor of 4 [24]) at a 23–44 GPa pressure along with essential intensity decreasing [25, 24].

There is an essential feature in Raman spectra of the the 2–5-nm nanodiamond sample under a 50 GPa pressure, namely, the absence of the 1325 cm^−1^ band, in spite of the intensity of this band even exceeding the intensity of the 1600 cm^−1^band. The Raman band of hydrostatically compressed diamond with a 443 GPa bulk modulus appears from under a singlet mode of a stressed diamond anvil [13] at pressure of at least 16 GPa [21]. The singlet mode *ω*_s_ of the stressed anvil tip depends on the pressure in the sample P_s_ as [13]

2 $$ \partial {\omega}_{\mathrm{s}}/\partial {\mathrm{P}}_{\mathrm{s}}=2.24{\mathrm{cm}}^{-1}/\mathrm{GPa} $$

while for the hydrostatically compressed diamond, the dependence is [21]

3 $$ \partial {\omega}_{\mathrm{d}}/\partial {\mathrm{P}}_{\mathrm{s}}=2.90\ {\mathrm{cm}}^{-1}/\mathrm{GPa} $$

Taking into account that *ω*_0_ = 1325 cm^−1^ in the relation (1) and after the simplest calculations from Eqs. (1–3), we could conclude that the absence of the band with *ω*_0_ = 1325 cm^−1^ under pressure of 50 GPa is possible only for a case when the bulk modulus of the 2–5-nm nanodiamond exceeds 524 GPa.

**Fig. 6:**
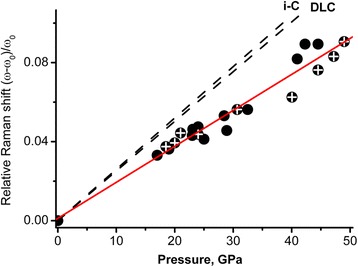
Dependence of the 1600 cm^−1^ relative Raman band shift upon pressure. Solid circles with crosses indicate a pressure increase; the ones without crosses belongs to a pressure decrease. A dash line reproduces dependences from Ref. [25] for diamond-like carbon DLC (in the Ref. [25] one marked as a-C) and glassy carbon i-C

As mentioned above, the 1600 cm^−1^ band belongs to the 2–5-nm nanodiamond. Consequently, we can estimate the bulk modulus using the pressure dependence of this Raman band plotted in Fig. 6. Solid circles with crosses belong to a pressure increase; the ones without crosses belong to a pressure decrease. Dash line reproduce the dependences from Ref. [25] for diamond-like carbon DLC (in the Ref. [25] marked as a-C) and glassy carbon i-C.

From a least-squares fit of the dependence in Fig. 6 for the 2–5-nm nanodiamond and Eq. (1), we obtain the bulk modulus of the 2–5-nm nanodiamond *B*_2-5nm_ = 564 GPa for *γ* ≈ 1, as mentioned above. For comparison, the dependence for DLC gives the 392 GPa bulk modulus for *γ* ≈ 1.

**Fig. 7:**
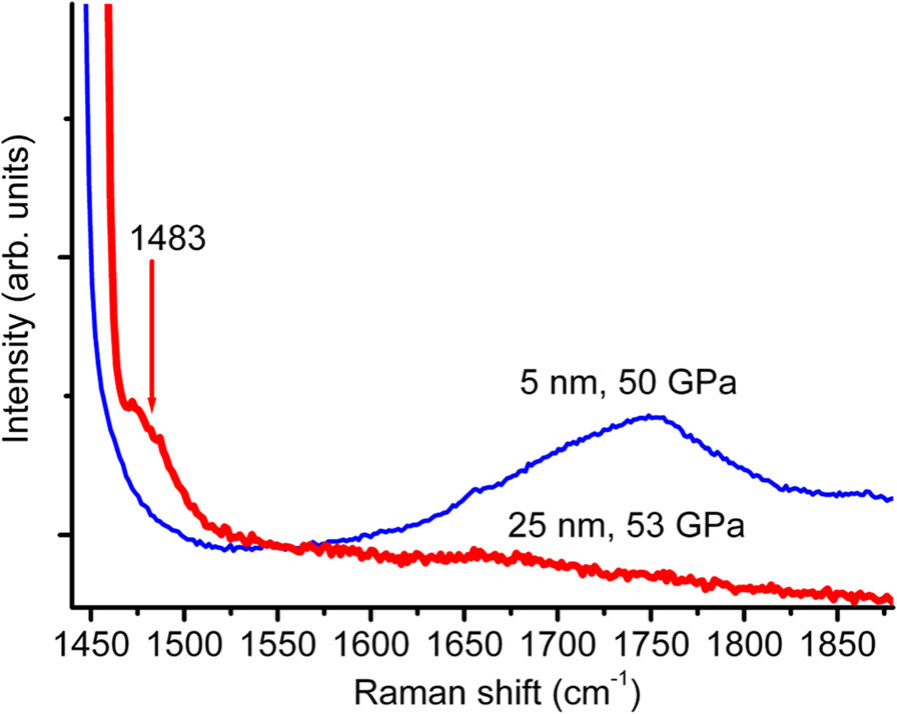
Raman spectra of the 25- and 2–5-nm nanodiamond under a 50 GPa pressure. Excitation wavelength is 458 nm. An initial 25-nm nanodiamond band 1329 cm^−1^ shifted to 1483 cm^−1^ exactly in accordance with the pressure dependence (2) of Raman mode of diamond with the bulk modulus 443 GPa. An additional band of the 25-nm nanodiamond around 1800 cm^−1^ showed a typical behavior for a G band of sp^2^-bonded carbon: disappearance of this band under pressure of 50 GPa

**Fig. 8:**
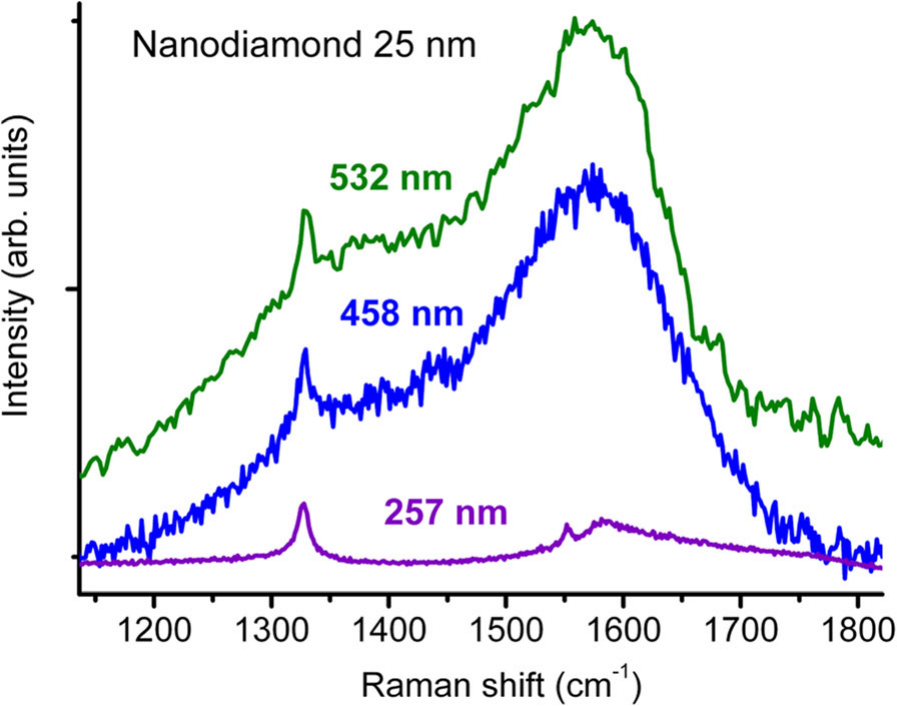
Raman spectra of the 25-nm nanodiamond. An additional band around 1580 cm^−1^ shows a typical behavior for a G band of the sp^2^-bonded carbon: the intensity decreases by a factor of 50–100 upon changing the excitation wavelength from 532/458 nm to 257 nm. A luminescence background is subtracted from the spectra with the excitation wavelength from 532/458 nm

All the experimentally observed features of the 2–5-nm nanodiamond (Raman bands 1325, 1500–1630, and 1600 cm^−1^, bulk modulus *B*_2-5nm_ = 564 GPa, preservation of the halfwidth and the intensity of the 1600 cm^−1^ band at least 50 GPa) we attribute, as mentioned above, to quantum confinement effect and the related increasing of nanodiamond bandgap. Consequently, these effects must disappear upon increasing of the nanodiamond size by a factor of 2–3 above the Bohr radius of exciton [2], that is above 10 nm. To check this supposition, a high-pressure study up to 53 GPa of the nanodiamond water suspension with a mean diamond crystal size of 25 nm was done. An initial 25-nm nanodiamond 1329 cm^−1^ band shifts to 1483 cm^−1^ exactly in accordance with the pressure dependence (2) of Raman mode of diamond with the 443 GPa bulk modulus (Fig. 7). A band around 1580 cm^−1^ shows a typical behavior for a G band of sp^2^-bonded carbon: the intensity decreases by a factor of 50–100 upon changing the excitation wavelength from 532/458 nm to 257 nm (Fig. 8), and disappearance of this band under pressure of 50 GPa. Consequently, the properties of the 25-nm nanodiamond are similar to those of common diamond contaminated with sp^2^-bonded carbon.

## Conclusions

Raman spectra of a 2–5-nm nanodiamond consist of 3 bands at 1325 cm^−1^, 1500–1630 cm^−1^ (depending on the excitation wavelength of 458–257 nm, accordingly), and 1600 cm^−1^. The 1600 cm^−1^ band cannot be attributed to a fraction of sp^2^-bonded carbon, because the intensity of this band does not depend on the excitation wavelengths of 458 and 257 nm (while the intensity of sp^2^-bonded carbon depends essentially on these wavelengths), and one halfwidth and the intensity do not change visibly under pressure at least up to 50 GPa (contrary to pressure-induced transformations of sp^2^-bonded carbon). The presence of the additional high-frequency (comparing to diamond) bands in the Raman spectra means an increase (comparing to diamond) in the elastic module according to dynamical theory of crystal lattices. The dependence of Raman spectra of the 2–5-nm nanodiamond upon pressure provides information on bulk modulus which we estimate as 564 GPa.

## References

[1] Mochalin VN, Shenderova O, Ho D, Gogotsi Y (2012). The properties and applications of nanodiamonds. Nat Nanotechnol.

[2] Gaponenko S (2010) Introduction to Nanophotonics. Cambridge University Press, Cambridge

[3] Belobrov PI, Bursill LA, Maslakov KI, Dementjev AP (2003). Electron spectroscopy of nanodiamond surface states. Appl Surf Sci.

[4] Fang X, Mao J, Levin EM, Schmidt-Rohr K (2009). Nonaromatic core-shell structure of nanodiamond from solid-state NMR spectroscopy. J Am Chem Soc.

[5] Chang YK, Hsieh HH, Pong WF, Tsai M-H, Chien FZ, Tseng PK (1999). Quantum confinement effect in diamond nanocrystals studied by X-ray-absorption spectroscopy. Phys Rev B.

[6] Gilman JJ (2002). Why diamond is very hard. Phil Magazine A.

[7] Pantea C, Zhang J, Qian J, Zhao Y, Migliori A, Grzanka E (2006). Nano-diamond compressibility at pressures up to 85 GPa. NSTI-Nanotech.

[8] Yur’ev GS, Dolmatov VY (2010). X-ray diffraction study of detonation nanodiamonds. Journal of Superhard Materials.

[9] Profeta M, Mauri F (2001). Theory of resonant Raman scattering of tetrahedral amorphous carbon. Phys Rev B.

[10] Ovsyannikov DA, Popov MY, Perfilov SA, Prokhorov VM, Kulnitskiy BA, Perezhogin IA (2017). High-hardness ceramics based on boron carbide fullerite derivatives. Phys Solid State.

[11] Ovsyannikov DA, Popov MY, Buga SG, Kirichenko AN, Tarelkin SA, Aksenenkov VV (2015). Transport properties of nanocomposite thermoelectric materials based on Si and Ge. Phys Solid State.

[12] Ponomarev OV, Popov MY, Tyukalova EV, Blank VD (2016). A ceramic nanocomposite with enhanced hardness based on corundum modified with carbon. Tech Phys Lett.

[13] Popov M (2004). Pressure measurements from Raman spectra of stressed diamond anvils. J Appl Phys.

[14] Daimay LV, Colthup NB, Fateley WG, Grasselli JG (1991). The handbook of infrared and Raman characteristic frequencies of organic molecules.

[15] Blank VD, Buga SG, Dubitsky GA, Serebryanaya NR, Sulyanov SN, Popov MY (1996). Phase transformations in solid C60 at high pressure-high temperature treatment and structure of 3D polymerized fullerites. Phys Lett A.

[16] Popov M, Mordkovich V, Perfilov S, Kirichenko A, Kulnitskiy B, Perezhogin I (2014). Synthesis of ultrahard fullerite with a catalytic 3D polymerization reaction of C60. Carbon.

[17] Ferrari AC, Robertson J (2004). Raman spectroscopy of amorphous, nanostructured, diamond-like carbon, and nanodiamond. Phil Trans R Soc Lond.

[18] Piscanec S, Mauri F, Ferrari AC, Lazzeri M, Robertson J (2005). Ab initio resonant Raman spectra of diamond-like carbons. Diam Relat Mater.

[19] Born M and Huang K (1954) Dynamical theory of crystal lattices. Oxford University Press, London

[20] Weinstein BA, Zallen R, Guntherodt MCG (1984). Pressure-Raman effects in covalent and molecular solids. Light scattering in solids.

[21] Hanfland M, Syassen K, Fahy S, Louie SG, Cohen ML (1985). Pressure dependence of the first-order Raman mode in diamond. Phys Rev B.

[22] Hanfland M, Beister H, Syassen K. (1989) Graphite under pressure: Equation of state and first-order Raman modes. Phys Rev B 39:12598 10.1103/physrevb.39.12598 9948126

[23] Meade C, Jeanloz R (1988). Yield strength of the B1 and B2 phases of NaCl. J Geophys Res.

[24] Goncharov AF, Makarenko IN, Stishov SM (1989) Graphite at pressure up to 55 GPa: optical properties and Raman scattering-amorphous carbon? Z. Exp Teor Fiz 96:670–673

[25] Goncharov AF, Andreev VD (1991) Raman scattering in carbon films at high pressures. Sov Phys JETP 73:140–142

